# The Measurement of Hemp Concrete Thermal and Moisture Properties for an Effective Building Construction Proposal in Region of Slovakia (Central Europe)

**DOI:** 10.3390/ma18071651

**Published:** 2025-04-03

**Authors:** Richard Hrčka, Patrik Štompf, Stanislav Jochim, Marek Eduard Mikuš, Milan Iskra

**Affiliations:** 1 Department of Wood Science, Faculty of Wood Sciences and Technology, Technical University in Zvolen, 960 01 Zvolen, Slovakia; 2 Department of Wooden Constructions, Faculty of Wood Sciences and Technology, Technical University in Zvolen, 960 01 Zvolen, Slovakia; xstompf@is.tuzvo.sk (P.Š.); jochim@is.tuzvo.sk (S.J.); xmikusm1@is.tuzvo.sk (M.E.M.); xiskra@is.tuzvo.sk (M.I.)

**Keywords:** hemp concrete, thermal properties, moisture properties, construction

## Abstract

The construction industry is facing an increased demand to adopt sustainable green building materials to minimize the carbon footprint. Hemp concrete is a green building material not only because of its low embodied carbon but also because of its ability to regulate heat and relative humidity. Its thermal characteristics are often viewed as favorable for reducing the energy used to heat or cool indoor buildings. The current research is focused on the properties of hemp concrete from Slovak manufacturers which can be effectively used in construction as a replacement for conventional building materials and can also be effectively applied in building renovations. The basic thermal properties of hemp concrete, i.e., specific heat capacity, thermal conductivity, effusivity, thermal diffusivity, and lag time, were determined. The determination of all properties is dependent on the knowledge of heat fluxes at the surface and the density of samples. The insulation ability was expressed with a thermal conductivity of 0.099 W·m^−1^·K^−1^. The accumulation was expressed with a specific heat of 1540 J·kg^−1^·K^−1^ and density of 322 kg·m^−3^ in the air environment temperature of 22 °C and relative humidity of 50%. To assess moisture properties, the moisture content and the speed of molecules during diffusion and lag time, based on the thickness of the hemp concrete samples, were measured. The speed of water molecules during diffusion in hemp concrete was 8.6 × 10^−7^ m·s^−1^. The study shows that hemp concrete has interesting hydrothermal properties for use as an insulation layer in envelope structures. Thus, this material can be used effectively in the construction field in order to meet the requirements of the current standards, which aim to reduce energy and environmental impacts.

## 1. Introduction

The construction sector is the largest consumer of resources and energy in Europe and is a major contributor to resource depletion and waste generation [1,2,3]. Therefore, attention in the sector is increasingly focused on renewable, nature-based, and locally available materials with low carbon emissions and embedded energy. Consequently, the demand for green construction materials and buildings will increase substantially in the coming years. The importance of using natural fiber materials has been extensively documented in papers that have focused on their physical and mechanical properties and their utilization in the building industry, e.g., [4,5,6,7,8].

Hemp concrete is a lightweight bio-based material, which attracts the attention of developers in Slovakia, e.g., [9,10,11]. This lightweight bio-based material is intended to be an excellent thermal insulator and moisture diffusor with enhanced durability [3,12]. It holds the potential to significantly impact the climate quality inside buildings and their overall sustainability from a life cycle perspective [7,8,13,14]. Hemp concrete consists of the inner woody core of the hemp plant mixed with a lime-based binder (hydraulic lime and non-hydraulic lime) and water [15,16].

Hemp hurd is typically obtained from the stalks of the hemp plant, which are harvested and processed to extract the fibers. The fibers are then cleaned and separated to remove any impurities, ensuring that they are ready for use in the production of hemp concrete [14,17]. Hydraulic lime consists of Ca(OH)_2_ (calcium hydroxide) and C_2_S (dicalcium silicates), also known as belit, which is reactive with moisture and undergoes hydration. The setting of calcic limes occurs through the uptake of carbon dioxide from the atmosphere with carbonation, forming stable calcium carbonate [5,16].

The structure of hemp concrete indicates its porous and heterogeneous character [18,19,20]. The natural plant origin of hemp indicates its hygroscopicity [21]. The porous character is represented by porosity [22], heterogeneity by the spatial distribution of density, anisotropy by the influence of measurement direction [23,24], and hygroscopicity by the equilibrium moisture content. All four properties are factors that influence the accumulating, insulation, or time lag character of hempcrete during the transport of heat, water, or air. The thermal properties are mass-specific heat capacity related to accumulation, thermal conductivity related to insulation, and thermal diffusivity related to time lag. The property related to the diffusion of water in hemp concrete is the velocity of constant concentration surface (isostere), which together with thickness is related to diffusivity (or diffusion coefficient).

Due to ongoing climate change, the construction industry is focusing more on the production of building materials based on natural raw resources [7,25]. Composites made from hemp hurds can be used both for the construction of new buildings and for the renovation of existing buildings, e.g., residential, office, and industrial buildings [26]. In building structures, hemp concrete has found application in several construction technologies, one of which includes formwork mounted on a wooden frame whose structure corresponds to the design layout of the building’s walls, floors, and roof [27,28]. It can be monolithic, sprayed, or precast in the case of hemp bricks or panels. First, it is poured between two formwork plates, and when the mold is placed, each layer is leveled to around 200 mm in thickness for each addition [29]. Hemp concrete with a density ranging from 250 to 350 kg·m^−3^ is commonly used to insulate exterior walls in low-rise construction projects and has been used in curtain walls for larger projects [20,30]. Hemp concrete is currently used to a relatively small extent in the Slovak region. Its applications are mainly limited to small buildings, various extensions, and residential buildings. From the point of view of its physical and environmental properties, hemp concrete appears promising even for use in various types of buildings under renovation as a main thermal insulation material.

The aim of the article is the measurement of heat capacity, thermal conductivity, thermal diffusivity, equilibrium moisture content, diffusion coefficient, density, and particle density of hemp concrete from the producer “LAUDATOSI—Healthy Hemp buildings” intended for applications within the Slovak region, or in the context of Central Europe. A further objective is to highlight the potential of hemp concrete for the renovation of existing office buildings based on previously measured data. The results of the research will be transformed into a theoretical design of a suitable wall assembly for the renovation of the Technical University in Zvolen’s office building with the aim of reducing energy consumption and improving indoor environmental quality.

## 2. Materials and Methods

**Figure 1:**
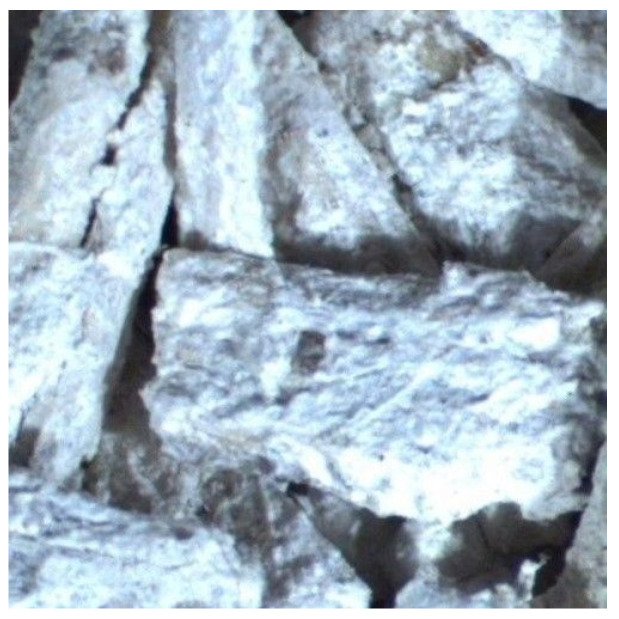
A microscopic picture of the hemp concrete cross-section (magnification 6×).

Hemp shives are crop by-products. The hemp shive aggregates supplied by manufacturers of hemp concrete originate from the Central European region. A commercially available pre-formulated hemp binder product was used in this research—a natural hydraulic lime and hydrated lime. In the investigation of thermal and moisture properties, it was necessary to prepare appropriate specimens of hemp concrete for subsequent testing under laboratory conditions. This research was supported by local Slovak hemp distributor “LAUDATOSI—Healthy Hemp buildings” (Banská Bystrica, Central Slovakia). The partner provided and finally processed the raw materials—hemp hurd and binder—into the final specimens (Figure 1). The studied materials are representative of the materials usually produced by the partner. To facilitate handling, the samples were placed into wooden frames.

The hemp hurds came from the E.U. area and they were part of technical hemp utilized for building purposes. The length of the hemp hurds ranged from 3 to 25 mm, with an average of 5 to 20 mm. The portion of hurds was 98% and dust particles max. 2%. The density of hemp hurds ranged from 100 to 110 kg·m^−3^ at a moisture content of 16%. For this study, hemp concrete was manufactured as rectangular specimens. The dimensions of the specimens were 86 × 86 × 11 mm. A total of 36 specimens were prepared according to the procedure of mixing 1:2 (110 kg of hemp hurd to 220 kg of hydraulic lime).

### 2.1. Thermal Properties and Density Measurement

**Figure 2:**
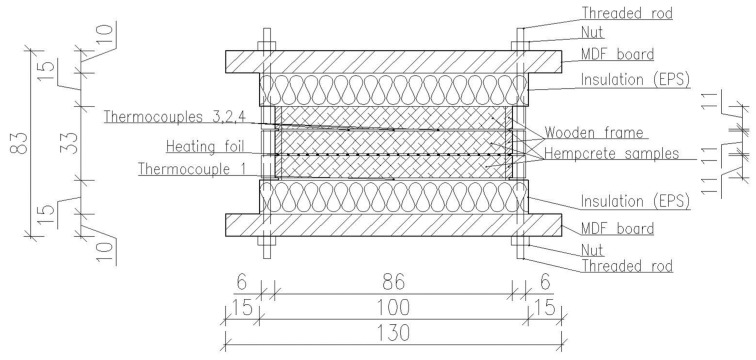
Measurement setup for measuring thermal properties.

Mass-specific heat capacity, thermal conductivity, and thermal diffusivity are thermal properties that can be measured simultaneously using transient methods. The transient methods are based on the solutions to the heat conduction equation. The methods are used to compare the solutions along with the measurement of temperature at various points in the specimens. The comparing criterion follows the method of least squares. The determination of all properties is dependent on knowledge of heat flux at the surface and the density of samples, considering that the particular solution of the heat conduction equation depends on initial and boundary conditions. The theoretical solution must meet the conditions and possibilities of the experimental apparatus, as shown in Figure 2.

The method of measuring hemp concrete thermal properties is based on a three-dimensional heat conduction equation in finite parallelepiped:

(1) $$\frac{\partial \vartheta }{\partial t}=a_{T}\frac{\partial^{2}\vartheta }{\partial x^{2}}+a_{R}\left(\frac{\partial^{2}\vartheta }{\partial y^{2}}+\frac{\partial^{2}\vartheta }{\partial z^{2}}\right)$$

and its transient solution in the form:

$$\vartheta \left[x,y,z,t\right]-\vartheta_{0}=8\frac{\varphi }{c\rho L}\sum_{k=1}^{24}\sum_{n=1}^{\infty }\frac{{\mathrm{Bi}}_{L}\sin\left[\mu_{n}\frac{L_{z}}{L}\right]+\mu_{n}\cos\left[\mu_{n}\frac{L_{z}}{L}\right]}{2{\mathrm{Bi}}_{L}+\mu_{n}^{2}+{\mathrm{Bi}}_{L}^{2}}\left(\mu_{n}\cos\left[\mu_{n}\frac{x}{L}\right]+{\mathrm{Bi}}_{L}\sin\left[\mu_{n}\frac{x}{L}\right]\right)$$

$$\sum_{p=1}^{\infty }\frac{\sin\left[\mu_{p}\left(\frac{4k-1.5}{45}\right)\right]-\sin\left[\mu_{p}\left(\frac{4k-2.5}{45}\right)\right]\cos\left[\mu_{p}\frac{y}{R}\right]}{\mu_{p}+\sin\left[\mu_{p}\right]\cos\left[\mu_{p}\right]}$$

(2) $$\sum_{s=1}^{\infty }\frac{\sin\left[\mu_{s}\right]\cos\left[\mu_{s}\frac{z}{T}\right]}{\mu_{s}+\sin\left[\mu_{s}\right]\cos\left[\mu_{s}\right]}\frac{\left(1-e^{-\left(a_{L}\frac{\mu_{n}^{2}}{L^{2}}+a_{R}\left(\frac{\mu_{p}^{2}}{R^{2}}+\frac{\mu_{s}^{2}}{T^{2}}\right)\right)t}\right)}{a_{L}\frac{\mu_{n}^{2}}{L^{2}}+a_{R}\left(\frac{\mu_{p}^{2}}{R^{2}}+\frac{\mu_{s}^{2}}{T^{2}}\right)}$$

where *θ*[*x*, *y*, *z*, *t*] is temperature at point [*x*, *y*, *z*] of coordinates and time *t*; *θ*_0_ is constant initial temperature; *φ* is flux at the surface *x* = 0 m; *c* is mass-specific heat capacity; *ρ* is density at given moisture content; *L* is length; *R* is width; *T* is thickness; *k* is the position of the strip in heating foil; (4*k* − 1.5) and (4*k* − 2.5) are the positions in mm of edges of a 1 mm thick strip; 45 is the half-width in mm of the whole heating foil, which consists of 12 strips with thickness of 1 mm and gaps between them with thickness of 3 mm; *L_z_* is the position of the source from surface *x* = 0 m; *Bi_L_* is Biot criterion; *μ_n_*, *μ_p_*, *μ_s_* are roots of characteristic equations.

The particular solution is derived using the Fourier transform method [31,32], fulfilling the constant initial condition and boundary conditions of the second and third kinds related to characteristic equations:

(3) $$cotg\left[\mu_{n}\right]=\frac{\mu_{n}^{2}-{Bi}_{L}^{2}}{2{Bi}_{L}\mu_{n}}$$

(4) $${Bi}_{R}=\mu_{p}tg\left[\mu_{p}\right]$$

(5) $${Bi}_{T}=\mu_{s}tg\left[\mu_{s}\right]$$

where *Bi_R_*, *Bi_T_* are Biot criteria. The time lag *FP* computing formula for a one-dimensional problem is:

(6) $$FP=\frac{L^{2}}{2a_{L}}$$

in the direction of the thickness *L*, and thermal diffusivity *a_L_* in the same direction.

The material of the source is a composition of nickel and chrome alloy known as “Vacronium” with a thickness of 0.01 mm arranged in a regular meander at the surface *x* = *L_z_* of the specimens. When an electric current passes through the meander, it converts electric energy into heat, which increases the temperature of the adjacent specimens. Temperature is measured with thermocouples of type K from Omega (Norwalk, CA, USA). The temperature increase in time is recorded with the datalogger Almemo 2890-9 (Holzkirchen, Germany), commonly used to record various quantities in transient processes. The QPX1200SP (Cambridgeshire, UK) source is used to produce a constant electric current. The increase in temperature at the source does not significantly influence the voltage between the ends of the heating foil. All measurements were performed inside a climatic chamber, Binder KBF 720 (Tuttlingen, Germany). The boundary conditions of the surrounding air—relative humidity of 50% and temperature of 20 °C—were maintained constantly inside the climatic chamber. Masses of samples were measured using a Kern KB 1000-2 scale (Balingen, Germany) until equilibrium was reached. Then, the density of the specimens was computed according to the formula:

(7) $$\rho =\frac{m}{abc}$$

where *m* is the mass of the sample; *a*, *b*, *c* are the dimensions of a rectangular sample. All dimensions of the sample were measured using the caliper Mitutoyo (Kanagawa, Japan).

### 2.2. Measurement of Water Molecules’ Translation Speed During Diffusion and Equilibrium Moisture Content

The concentration of mass water molecules in a volume of oven-dried specimens is the transport potential of water molecule diffusion in a volume of hemp concrete specimens. The driving force is the gradient of this concentration. The solutions of the diffusion equation are as follows:

(8) $$\frac{\partial c}{\partial t}=\frac{\partial }{\partial x}\left(D\frac{\partial \vartheta }{\partial x}\right)$$

which describes the changes in concentration *c* in space *x* and time *t*. For a constant concentration:

(9) $$0=\frac{\partial c}{\partial t}+v\frac{\partial c}{\partial x}$$

where speed *v* of the constant concentration is independent of space coordinate *x*.

After comparing Equations (6) and (7), the diffusivity and speed are related:

(10) $$D=v\left(L-x\right)$$

because *D* is zero at *x* = *L*. Then, the one-dimensional transient diffusion equation has the form:

(11) $$\frac{\partial c}{\partial t}=\frac{v}{4}\left(\frac{\partial^{2}c}{\partial \xi^{2}}+\frac{1}{\xi }\frac{\partial c}{\partial \xi }\right)$$

where *ξ* = (*L* − *x*)^0.5^. The particular solution of Equation (9) fulfills the variable boundary condition of the second kind at surface *x* = 0 m:

(12) $$\frac{\partial \bar{c}}{\partial t}={D\left.\frac{\partial c}{\partial x}\right|}_{x=0}$$

where $\bar{c}$ is the average concentration:

(13) $$\bar{c\left[t\right]}=\frac{1}{L}\int_{0}^{L}c\left[x,\;t\right]dx$$

There is zero flux at the rear surface of the specimen, *x* = *L*. The initial condition is a constant concentration through the thickness. Then, a particular solution is in the form:

(14) $$c\left(x,t\right)=\bar{c}+\sum_{k=1}^{\infty }\frac{J_{0}\left[\mu_{k}\sqrt{1-\frac{x}{L}}\right]}{J_{0}\left[\mu_{k}\right]}e^{-\frac{\mu_{k}^{2}vt}{4L}}\int_{0}^{t}\frac{\partial \bar{c}}{\partial \tau }e^{\frac{\mu_{k}^{2}v\tau }{4L}}d\tau$$

where *µ_k_* is the *k*-root of the characteristic equation *J*_1_(*µ*) = 0.

This particular solution indicates the need to measure the average concentration at the time of diffusion. Then, the solution describes the concentration field inside the specimens as always fulfilling the average concentration. To complete the solution, one needs to know the value of the water molecules’ translation speed. The value is determined as the maximum value of all speeds along with the maximum value of the surface concentration, which does not exceed the equilibrium concentration at the beginning of diffusion. Equilibrium at the surface is reached at time *t_max_*, corresponding to the largest slope of average concentration change from a beginning value of zero against the square root of time.

Then, speed is determined according to the formula:

(15) $$v=\frac{2}{3}L\frac{{\left.\frac{\partial c}{\partial t}\right|}_{t=t_{max}}}{\left(c_{\infty }-{\bar{\left.c\right|}}_{t=t_{max}}\right)}=\frac{2}{3}L\frac{{\left.\frac{\partial c}{\partial t}\right|}_{t=t_{max}}}{\sum_{k=1}^{\infty }e^{-\frac{\mu_{k}^{2}vt_{max}}{4L}}\int_{0}^{t_{max}}\frac{\partial \bar{c}}{\partial \tau }e^{\frac{\mu_{k}^{2}v\tau }{4L}}d\tau }$$

where *c*_∞_ is the equilibrium concentration. The division of the equilibrium concentration by density results in the equilibrium moisture content. Measurement of the equilibrium moisture content was performed every 10 min on tared Radwag PS 600.R2 scales (Radom, Poland) connected to a computer for storing the measured data. The experiment was performed in a climatic chamber, Binder KBF 720. The boundary conditions of surrounding air—relative humidity of 50% and temperature of 20 °C—were maintained constantly inside the climatic chamber.

### 2.3. Measurement of Bound Water Maximum Moisture Content and Density of Particles

The measurement of bound water maximum moisture content was performed in a water environment with a temperature of 20 °C using the method described in [33]. Individual particles of hemp concrete were weighed with a Radwag XA 60/220/X scale (Radom, Poland) using a density determination kit in a water environment, and mass *m_z_* was recorded in equilibrium. This mass is the maximum bound water mass of the specimen, which is equal to the maximum bound water mass of particles. The maximum mass of particles was measured in an environment of moist air with relative humidity of 100% and temperature of 20 °C. The difference between the maximum mass and oven-dried mass of individual particles is the total water mass of particles. The maximum mass of particles in free water is the difference between the total water mass and the maximum bound water mass of particles. The bound water maximum moisture content is the ratio:

(16) $$w_{Bmax}=\frac{m_{z}}{m_{0}}$$

where *m*_0_ is oven-dried mass. The oven-dried state was achieved in an environment of dry air with a temperature of 105 °C until constant mass was recorded. The density of individual particles *ρ_particle_* is:

(17) $$\rho_{particle}=\rho_{H_{2}O}\frac{m_{max}}{m_{max}-m_{z}}$$

where *m_max_* is the maximum mass of particles. The oven-dried densities of specimens were measured in an oven-dried state using Equation (7).

### 2.4. Calculation of Thermal and Moisture Properties of Proposed Assemblies

The thermal and moisture properties of two proposed structural assemblies of external walls for the renovation of the existing office building at the Technical University in Zvolen were calculated using the Fragment program. The calculation program is designed for the basic thermal and moisture assessment of fragments of envelope structures under one-dimensional heat and moisture conduction. The internal boundary conditions of the calculation were set according to the standard STN [34], which defines basic boundary conditions for internal rooms of residential buildings. The external boundary conditions based on climatic data available from long-term measurements for the city of Zvolen (central Slovakia) were also included in the calculation program. The basic physical properties of materials used in the calculation were set according to the manufacturer’s technical data sheets. For hemp concrete, we used physical properties that were found in the research.

## 3. Results

### 3.1. Thermal Properties of Hemp Concrete

The accumulation property of a material is represented by heat capacity. The ultimate properties representing accumulation are mass-specific heat capacity or volume-specific heat capacity. Both essential properties relate to density. The insulation property is represented by thermal conductivity. Lower thermal conductivity results in better insulation properties during conduction. The time lag property is represented by thermal diffusivity, which is proportional to the speed of equilibrating two different temperatures in a specimen during conduction. Larger thermal diffusivity results in a shorter time lag. The effusivity characterizes the contact temperature between two solids during conduction. Larger effusivity results in a closer surface temperature to the initial temperature. The hemp concrete thermal properties are shown in Table 1.

**Figure 3:**
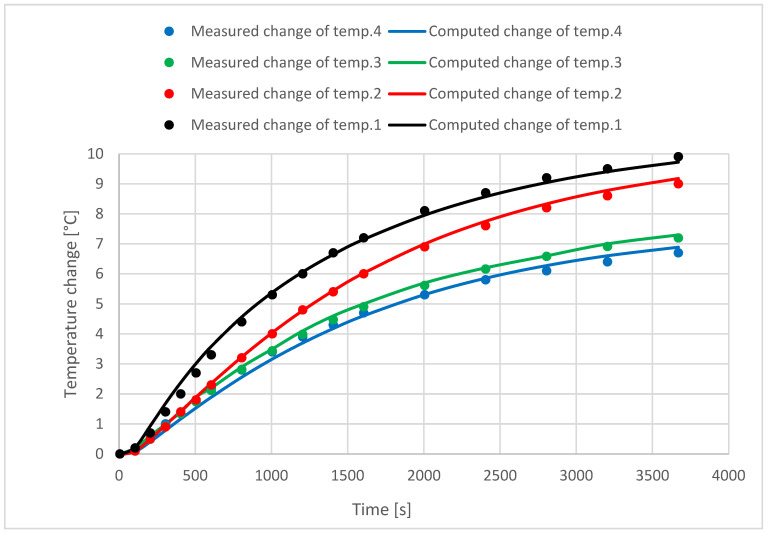
Measured and computed temperatures at various positions in hemp concrete specimens.

The results in Table 1 come from measurements using the apparatus described in Section 2. The measured temperature differences from the initial temperature of 22 °C for different positions of thermocouples are shown in Figure 3 in the form of dots.

The computed temperature differences are depicted as lines on the base of results in Table 1 and Equation (2). All results fulfill simultaneously the least squares method criterion. A stop time of 3600 s was required to compute the results.

### 3.2. Moisture Properties of Hemp Concrete

The same analysis can be performed with water inside hemp concrete specimens during diffusion. The accumulation property is characterized by equilibrium moisture content as an intensive property. The insulation property is represented by the diffusion coefficient or diffusivity. And time lag is inversely proportional to the speed of molecules during diffusion. The results in Table 2 come from measurements taken using the apparatus described in Section 2.

**Figure 4:**
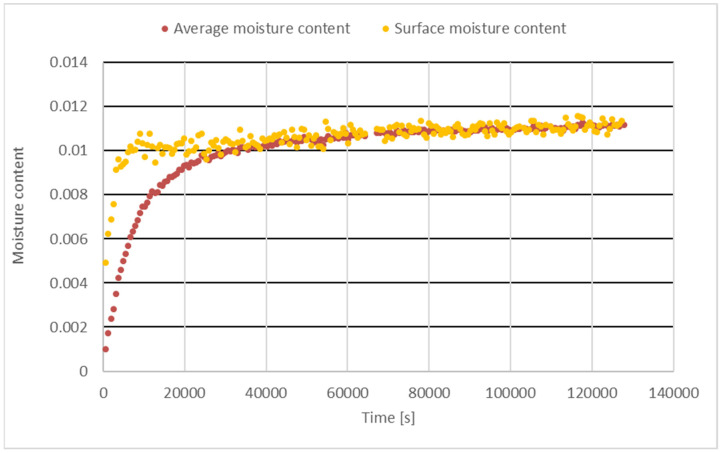
Measured average and computed surface moisture content of hemp concrete.

The measured average moisture content and computed surface moisture content are shown in Figure 4.

The surface moisture content does not exceed the equilibrium moisture content.

The equilibrium moisture content of hemp concrete was 1.2% in the air environment with relative humidity of 20% and temperature of 20 °C. The oven-dried density and properties of hemp concrete, related to soaking in water, are shown in Table 3. The results shown in Table 3 were measured using the Archimedes principle.

### 3.3. Design of Hemp Concrete Wall Composition

A further objective is to highlight the potential of hemp concrete for the renovation of the university’s existing office building based on previously measured data. The previous results of the research are transformed into a theoretical design of a suitable wall composition for the renovation of the Technical University in Zvolen’s office building with the aim of reducing energy consumption and improving indoor environmental quality and environmental impact.

**Figure 5:**
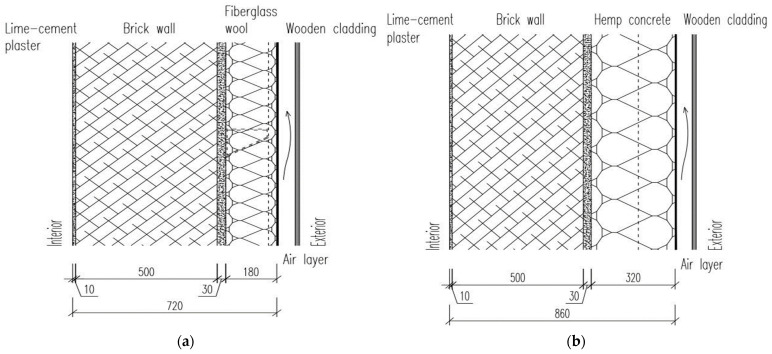
Investigated wall assemblies: (**a**) composition with fiberglass wool; (**b**) composition with hemp concrete.

The proposal to use hemp concrete as thermal insulation for the renovation of the administrative building is based on its physical, fire safety, and environmental properties. The use of hemp concrete in building renovation is also suitable due to the presence of concrete consoles on each story of the building, which can be effectively used as a supporting base element for the hemp concrete insulation layer. Two types of renovated wall assemblies of the building envelope, as described below and shown in Figure 5, were designed and subjected to a theoretical analysis of thermal and moisture properties:

- Original assembly: Lime–cement plaster (10 mm) + perforated bricks (500 mm) + external perlite plaster (30 mm) + ventilated air cavity and external wooded cladding (spruce); total thickness: 540 mm.
- Proposed Assembly 1: Lime–cement plaster (10 mm) + perforated bricks (500 mm) + external perlite plaster (30 mm) + fiberglass insulation boards inserted into the steel grid for external stone cladding (180 mm); total thickness: 720 mm.
- Proposed Assembly 2: Lime–cement plaster (10 mm) + perforated bricks (500 mm) + external perlite plaster (30 mm) + hemp concrete with integrated timber (or steel) grid for stone cladding (320 mm); total thickness: 860 mm.

Both assemblies were designed to meet the current thermal requirements according to the standard STN [34], particularly regarding the thickness of the thermal insulation layer. The hemp concrete thickness of 320 mm was selected to fulfill the requirement for a U-value of 0.22 W·m^−2^·K^−1^ according to Slovak technical norm STN 73 0540-2: Z2/2019 [34]. Table 4 shows the physical properties of the construction materials used in the design of the assemblies for calculating the thermal properties of the proposed walls. The diffusion resistance (η) of materials for the assemblies was necessary to investigate the potential of the maximum amount of condensed water vapor inside the proposed assemblies according to the standard STN [34].

Table 5 contains the results of the calculation of the thermal and moisture properties of the proposed assemblies of the building’s perimeter wall according to STN [34].

Both proposed assemblies meet the requirements of the standard STN [34] regarding the heat transfer coefficient (U-value) and the maximum amount of condensed water vapor inside the structures. During the simulated time, there was no condensation of water vapor inside the structures. The phase shift (Ψ) in the proposed Assembly 2 using hemp concrete is significantly higher (difference of 12.61 h) than in Assembly 1. These results indicate that the use of hemp concrete as the main insulating layer, compared to mineral wool, ensures better indoor environmental conditions and a slower reaction of the indoor environment to changes in outdoor temperature. These differences will be reflected in the overall heating demand in winter and cooling in summer.

## 4. Discussion

The comparison of results with literature data indicates the reliability of the mixing procedure for hemp concrete production in Slovakia. The results of hemp concrete thermal properties are within the data ranges shown in [3]. Reference [3] summarized results from different literature sources. The measurement of all three thermal properties simultaneously is enabled using the transient method. It seems better and faster in comparison to using a method with steady flux in the specimen or a transient method without knowing the flux at the surface [35]. The results of the surface moisture content shown in Figure 3 revealed that the whole body of hemp concrete specimens responded to the changing environmental parameters. The surface moisture content attained equilibrium gradually. The comparison of time lag results for heat conduction and water diffusion reveals that equilibration of the temperatures is reached 50 times faster compared to concentrations within hemp concrete. This indicates the possibility of investigating both processes separately at normal boundary conditions with a temperature of 20 °C and humidity of 50%. Equation (2) must fulfill the condition of continuum. If not, for heterogeneous regions, it must be used separately and fulfill the boundary conditions. But such a computation is time-consuming, and it does not perform well. Worth noting is the ability to store bound water inside the structure of hemp concrete. Its value of 56% is almost doubled in comparison to the bound water maximum moisture content for wood. Hemp concrete possesses the ability to store water inside its structure, especially at higher relative humidity. This property enables a more pleasant indoor environment in comparison to concrete, especially in the dry and cold winter season.

Previous studies have shown that the properties of hemp concrete (physical, mechanical) depend on various factors [36]. Significant characteristics of hemp concrete that affect its thermal performance and moisture properties include the particle size of hemp hurds, moisture content of the shives, proportions of hemp hurds to binder, binder mix design, mixing methods for the entire composition, placing, and compaction level [37,38]. All thermal properties of hemp concrete samples were measured at a relative humidity of 50% and a temperature of 22 °C.

Thermophysical properties are generally dependent on density. The average density (ρ) of 36 sample pieces was 322 kg·m^−3^, ranging from 302 to 345 kg·m^−3^. In [39], the wet density ranged from 304 to 336 kg·m^−3^, and the average was at the same level of 322 kg·m^−3^ as our samples. The density of hemp concrete is typically far lower than that of standard concrete aggregates. Hemp concrete has a wide range of densities, as was demonstrated by previous studies, e.g., [40,41]. The resulting density of hemp concrete depends on the individual component’s ratio in the given mixture, level of moisture content, and extent of compaction. In addition, the density of hemp concrete depends on the method of application in the structures.

The specific heat capacities (c) of wet samples range from 1330 to 1740 J·kg^−1^·K^−1^ with averages of 1535 J·kg^−1^·K^−1^. Previous studies showed that the specific heat capacity of hempcrete spans a wide range of values. Specific heat capacity values of hemp concrete for various densities recorded in different publications ranged from 183 to 1700 J·kg^−1^·K^−1^ [22,23,42,43]. Nowadays, improved specific heat is related to phase change materials (PCM). Such PCM enhances hemp concrete’s mass-specific heat capacity due to latent heat and phase conversion [44].

The thermal conductivity (λ) of the hemp concrete samples perpendicular to the plane of the samples (as shown in Table 1) ranged from 0.070 to 0.110 W·m^−1^·K^−1^, with an average of 0.099 W·m^−1^·K^−1^. The thermal conductivity of hemp concrete, as a basic thermophysical property, is influenced by several factors. Studies [24,45] have shown that the fiber orientation, the density of hemp concrete, and the method of mixing also affect thermal conductivity. According to the study [36], a higher proportion of lime in a hemp concrete mixture leads to higher thermal conductivity and specific heat capacity. However, research [46] suggests that increasing the hydraulic content of the binder reduces thermal conductivity and increases the heat capacity. Various studies reported that the dry thermal conductivity of the hemp concrete ranged from 0.05 W·m^−1^·K^−1^ to 0.138 W·m^−1^·K^−1^ [22,37,40,46]. Our data are within this range. Hemp concrete owes its excellent thermal conductivity properties to hemp hurd’s high porosity [47,48,49]. Organic binders, such as sapropel, can also be used to achieve lower thermal conductivity [50]. A recent investigation of hemp concrete with a silica sol binder [51] indicated that it has a thermal conductivity of 0.05 W·m^−1^·K^−1^, similar to hemp hurds, while preserving mechanical strength [52]. Thermal conductivity rises almost linearly with water content, according to [53,54].

According to the data in Table 1, the thermal effusivity (e) of hemp concrete samples ranged from 189 to 248 W·m^−2^·K^−1^·s^0.5^, with an average of 221 W·m^−2^·K^−1^·s^0.5^. In the research [55], the thermal effusivity value of hemp concrete was recorded at 231 W·m^−2^·K^−1^·s^0.5^. The results presented in the research [56] showed that the thermal effusivity of a hemp concrete wall is relatively low compared to most common masonry materials, but it is higher than classical insulation materials.

Understanding thermal diffusivity (a) was crucial for calculating the phase shift or lag time of the samples studied according to the applied methodology. The thermal diffusivity of the wet samples ranged from 1.5 to 2.3 × 10^−7^ m^2^·s^−1^, with an average of 2.0 × 10^−7^ m^2^·s^−1^. According to [13], the thermal diffusivity of the wet samples ranged from 1.893 to 2.069 × 10^−7^ m^2^·s^−1^, whereas that of dry specimens ranged from 2.076 to 2.321 × 10^−7^ m^2^·s^−1^, with averages of 1.970 × 10^−7^ m^2^·s^−1^ and 2.182 × 10^−7^ m^2^·s^−1^, respectively. Thermal diffusivity values reported in the literature [35,39,46,50] ranged between 0.18 and 1.68 × 10^−7^ m^2^·s^−1^.

One of the indicators of thermal properties is time lag. It defines the time of one-dimensional conduction of the isotherm from warmer to colder surfaces based on surface thickness. The computed time lag (or phase shift) of the samples (11 mm thick) ranged from 258 to 401 s, with an average of 301 s (Table 1). In the research [57], it is possible to observe that the increase in insulation layer thickness leads to an increase in time lag. In another study [58], this was similarly observed. In summer conditions, the time lag is important and should be increased to shift daily outdoor conditions’ propagation to the night when outdoor air is colder, and night cooling is efficient [59]. The research [55] compared hemp concrete with other common building materials (e.g., solid brick, concrete). In the study, it was proven that hemp concrete has the lowest thermal diffusivity and the longest time lag, which means that it can better reduce the propagation of outdoor weather conditions through building envelopes.

Hemp concrete is considered a green building material not only because of its low embodied carbon but also because of its ability to regulate heat, moisture, and relative humidity. Being highly porous, hemp hurds exhibit excellent water absorption behavior, up to 56% of bound water moisture content. The test results of previous research showed that hurds are capable of absorbing water equivalent to two-thirds of their own weight within 10 mm of immersion, reaching a saturation level of 95% [60,61,62,63]. Hemp concrete quickly equilibrates moisture content using both mechanisms of diffusion or permeability, for example, in comparison to wood. Large diffusivity and water vapor permeability were determined as nearly constant for low to mid relative humidity [50,64,65]. In addition, hemp concrete’s hygroscopic nature allows it to effectively regulate indoor humidity by absorbing and releasing water. The ability of a substance to modify ambient relative humidity is proportional to its moisture buffer value [66]. A recent study [62] analyzed hemp fibers and hurds’ equilibrium moisture content and heat adsorption. Both the hemp fibers and hemp hurds showed a hysteresis loop where the rate of water adsorption (0.57–0.66) was higher than the desorption rate (0.27–0.35) at a relative humidity range of 80–90%. This means that at a high relative humidity range, hemp hurds must adsorb water at a quicker rate compared to the rate of release during desorption [60]. The overall findings suggest that hempcrete mixes with a 1:1 binder-to-hemp-hurd ratio and 300–400 kg∙m^−3^ density have hygrothermal properties suitable for infill wall applications [13].

The assessment of proposed assemblies of envelope structures in the one-dimensional calculation program Fragment (as shown in Table 5) showed that using hemp concrete as a main insulation layer provides an appropriate solution for the renovation of the technical university’s office building. Both proposed assemblies meet the requirements of the standard STN [34] regarding the heat transfer coefficient and the maximum amount of condensed water vapor inside structures. The disadvantage of Assembly 2 (with hemp concrete) compared to Assembly 1 (with mineral wool) is the need for a significantly greater thickness of hemp concrete (a 320 mm hemp concrete layer compared to 180 mm for mineral wool), which is related to its thermal properties. However, in our specific case (university building renovation), the use of hemp concrete as the main thermal insulating layer is suitable due to the presence of concrete consoles on each story, which would be appropriately used as a base for this type of insulation. The results in Table 5 indicate that the use of hemp concrete as the main insulating layer, compared to mineral wool, ensures better indoor environmental conditions and a slower reaction of the indoor environment to changes in outdoor temperature. This represents the differences in the values of phase shift (Ψ) and thermal damping factors (ν). The phase shift was 21.15 h in Assembly 1 and 33.76 h in Assembly 2. The thermal damping factor in Assembly 2 (composition with hemp concrete) is almost 7.5 times higher than in Assembly 1 (with mineral wool). Fiberglass insulation is more suitable because of its lower thickness and cheaper cost in comparison to hemp concrete, but fiberglass is not a hygroscopic material. Hemp concrete is a hygroscopic material with the ability to redistribute water in the specimen volume and release it into a ventilated air gap.

## 5. Conclusions

Despite hemp concrete’s excellent thermal and moisture properties, its utilization in the construction industry remains low in the Slovak region due to the shortage of hemp, which is not commonly grown in the country at present. The company “LAUDATOSI—Healthy Hemp buildings” obtains hemp hurds from the surrounding countries of Central Europe and from Italy. The hemp concrete company HempTech Monolitics is currently starting its own hemp production. Attention in the Slovak region is being given to the use of hemp-based materials in construction due to their excellent thermal, moisture, and environmental properties. The gradual development of local cultivation also helps to improve the environmental characteristics. Consequently, this study provides valuable information about the thermal and moisture properties of hemp concrete made in Slovakia with hemp fibers originating in Central Europe. The study also highlights the potential of using hemp concrete not only in the construction of new buildings but also in renovations of administrative buildings where it has not been used thus far. The results of the study were transformed into a theoretical design of a suitable wall assembly for the renovation of the Technical University in Zvolen’s office building (Central Slovakia) with the aim of reducing energy consumption and improving indoor environmental quality. The research is likely to interest audiences, in academia and industry, who are focused on sustainable, environmentally friendly, and low-carbon composite building materials. The main conclusions of this study are as follows:

- Thermophysical properties are generally dependent on density. The average density (ρ) of hemp concrete specimens was 322 kg·m^−3^, ranging from 302 to 345 kg·m^−3^. The resulting density of hemp concrete depends on the individual component’s ratio in the given mixture, level of moisture content, and extent of compaction. In addition, the density of hemp concrete depends on the method of application in the structures. All thermal properties of hemp concrete samples were measured at a relative humidity of 50% and a temperature of 22 °C.
- The specific heat capacity (c) of wet hemp concrete samples ranged from 1330 to 1740 J·kg^−1^·K^−1^ with an average of 1535 J·kg^−1^·K^−1^.
- The thermal conductivity (λ) of the hemp concrete samples perpendicular to the plane of the samples ranged from 0.070 to 0.110 W·m^−1^·K^−1^, with an average of 0.099 W·m^−1^·K^−1^.
- According to the data, the thermal effusivity (e) of hemp concrete ranged from 189 to 248 W·m^−2^·K^−1^·s^0.5^, with an average of 221 W·m^−2^·K^−1^·s^0.5^. The thermal effusivity of hemp concrete walls is relatively low compared to most common masonry materials, but it is higher than classic insulation materials.
- Understanding the thermal diffusivity (a) was crucial for calculating the lag time (or phase shift) of the samples studied according to the applied methodology. The thermal diffusivity of the wet samples ranged from 1.5 to 2.3 × 10^−7^ m^2^·s^−1^, with an average of 2.0 × 10^−7^ m^2^·s^−1^.
- As one of the indicators of thermal properties, time lag defines the rate at which heat is absorbed and released by construction material. The computed time lag of the samples (11 mm thick) ranged from 258 to 401 s, with an average of 301 s.
- Hemp, as a bio-based material, has a porous anatomical structure and hygroscopic nature; thus, it can adsorb and desorb large amounts of water. The results of surface moisture content revealed that the whole body of hemp concrete specimen acted in response to the changing environmental parameters. The surface moisture content attained equilibrium gradually. The comparison of time lag results for heat conduction and water diffusion reveals it was 50 times faster in equilibrating the temperatures than concentrations within hemp concrete. This indicates the possibility of investigating both processes separately at normal boundary conditions with a temperature of 20 °C and humidity of 50%. Hemp concrete possesses the ability to store water inside its structure, especially at higher relative humidity. This property enables a more pleasant indoor environment in comparison to concrete, especially in the dry and cold winter season.
- Both proposed assemblies (with mineral wool or with hemp concrete) meet the requirements of the standard STN [38] regarding the heat transfer coefficient (U-value) and the maximum amount of condensed water vapor inside the structures. During the simulated time, there was no condensation of water vapor inside the structures. The phase shift (Ψ) in the proposed Assembly 2 using hemp concrete, unlike Assembly 1 with mineral wool, is significantly higher (difference of 12.61 h). These results indicate that the use of hemp concrete as the main insulating layer, compared to mineral wool, ensures better indoor environmental conditions and a slower reaction of the indoor environment to changes in outdoor temperature. These differences will be reflected in the overall heating demand in winter and cooling in summer.

## Figures and Tables

**Table 1:** The density ρ, mass-specific heat capacity c, thermal conductivity λ, volume-specific heat capacity c.ρ, effusivity e, thermal diffusivity a, and lag time at thickness h of hemp concrete.

	h (mm)	ρ (kg∙m^−3^)	c (J∙kg^−1^∙K^−1^)	λ (W∙m^−1^.K^−1^)	c∙ρ (J∙m^−3^∙K^−1^)	e (W∙m^−2^∙K^−1^∙s^0.5^)	a (m^2^∙s^−1^)	Lag Time (s)
average	11.0	322	1540	0.099	494,000	221	2.0 × 10^−7^	302
min		302	1330	0.070	422,000	189	1.5 × 10^−7^	258
max		345	1740	0.110	550,000	248	2.3 × 10^−7^	401
std. dev.		16	122	0.014	37,300	19	2.5 × 10^−8^	43
var. coef. (%)		5.1	7.9	14	7.6	8.6	13	14

No. of obs. 9.

**Table 2:** The speed of molecules during diffusion v and lag time at thickness h of hemp concrete specimens.

	H (mm)	v × 10^−7^ (m·s^−1^)	Lag Time (hours)
average	11.0	8.6	3.7
var. coef. (%)		30	27

No. of obs. 3.

**Table 3:** Oven-dried density of specimen ρ_0_, particle density at maximum moisture content ρ_particle_, and maximum moisture content of bound w_Bmax_ and free water w_Fmax_ of hemp concrete.

	ρ_0_ (kg·m^−3^)	ρ_particle_ (kg·m^−3^)	w_Bmax_ (%)	w_Fmax_ (%)
average	278	1300	56	85
var. coef. (%)	1.4	2.4	1.9	21

No. of obs. 4.

**Table 4:** Physical properties of envelope components for designed assemblies.

Material	Density ρ (kg∙m^−3^)	Thermal Conductivity λ (W∙m^−1^∙K^−1^)	Specific Heat Capacity c (J∙kg^−1^∙K^−1^)	Diffusion Resistance η (-)
Lime–cement plaster	2000	0.99	790	19
Perforated bricks	1400	0.61	960	7
Perlite plaster	400	0.12	850	11
Fiberglass board	23	0.036	940	1
Hemp concrete	322	0.10	1 435	10.17
Timber frame (softwood)	400	0.13	2 510	157
Chrome-alloyed steel (frame for external cladding)	7850	19	540	100,000
Vapor-permeable membrane	1000	0.21	1400	50

**Table 5:** The results of the calculation of the thermal and moisture characteristics of the proposed assemblies.

Assembly	Heat Transfer Coefficient U (W∙m^−2^∙K^−1^)	Thermal Resistance R (m^2^∙K∙W^−1^)	Water Vapor (kg∙m^−2^∙Year^−1^)		Phase Shift of Thermal Oscillation Ψ (h)	Thermal Damping Factor ν (-)
			Annual Amount of Condensed Water Vapor	Balance of Annual Amount of Condensed and Evaporated Water Vapor		
Assembly 1 (with fiberglass insulation)	0.219	4.355	- ^(1)^	- ^(1)^	21.15	1993.52
Assembly 2 (with hemp concrete)	0.224	4.247	- ^(1)^	- ^(1)^	33.76	14,854.47

Note: ^(1)^ no amount of condensed water vapor, without condensation inside the structures

## Data Availability

The original contributions presented in this study are included in the article. Further inquiries can be directed to the corresponding author.
